# Differential impact of body mass index and leptin on baseline and longitudinal positron emission tomography measurements of the cerebral metabolic rate for glucose in amnestic mild cognitive impairment

**DOI:** 10.3389/fnagi.2022.1031189

**Published:** 2022-11-22

**Authors:** Christopher M. Weise, Kewei Chen, Yinghua Chen, Vivek Devadas, Yi Su, Eric M. Reiman

**Affiliations:** ^1^Department of Neurology, Marti-Luther-University of Halle-Wittenberg, Halle, Germany; ^2^Department of Neurology, University of Leipzig, Leipzig, Germany; ^3^Banner Alzheimer’s Institute, Phoenix, AZ, United States; ^4^School of Mathematics and Statistics, Arizona State University, Tempe, AZ, United States; ^5^Department of Neurology, College of Medicine, University of Arizona, Phoenix, AZ, United States; ^6^Arizona Alzheimer’s Consortium, Phoenix, AZ, United States; ^7^School of Computing and Augmented Intelligence, Arizona State University, Tempe, AZ, United States; ^8^Department of Psychiatry, College of Medicine, University of Arizona, Phoenix, AZ, United States; ^9^Neurogenomics Division, Translational Genomics Research Institute, Phoenix, AZ, United States; ^10^Arizona State University-Banner Health Neurodegenerative Disease Research Center, Arizona State University, Tempe, AZ, United States

**Keywords:** BMI, leptin, FDG-PET, regional cerebral metabolic rate for glucose (rCMRgl), mild cognitive impairment (MCI)

## Abstract

**Introduction:**

Several studies have suggested that greater adiposity in older adults is associated with a lower risk of Alzheimer’s disease (AD) related cognitive decline, some investigators have postulated that this association may be due to the protective effects of the adipose tissue-derived hormone leptin. In this study we sought to demonstrate that higher body mass indices (BMIs) are associated with greater baseline FDG PET measurements of the regional cerebral metabolic rate for glucose (rCMRgl), a marker of local neuronal activity, slower rCMRgl declines in research participants with amnestic mild cognitive impairment (aMCI). We then sought to clarify the extent to which those relationships are attributable to cerebrospinal fluid (CSF) or plasma leptin concentrations.

**Materials and methods:**

We used baseline PET images from 716 73 ± 8 years-old aMCI participants from the AD Neuroimaging Initiative (ADNI) of whom 453 had follow up images (≥6 months; mean follow up time 3.3 years). For the leptin analyses, we used baseline CSF samples from 81 of the participants and plasma samples from 212 of the participants.

**Results:**

As predicted, higher baseline BMI was associated with greater baseline CMRgl measurements and slower declines within brain regions preferentially affected by AD. In contrast and independently of BMI, CSF, and plasma leptin concentrations were mainly related to less baseline CMRgl within mesocorticolimbic brain regions implicated in energy homeostasis.

**Discussion:**

While higher BMIs are associated with greater baseline CMRgl and slower declines in persons with aMCI, these associations appear not to be primarily attributable to leptin concentrations.

## Introduction

Estimations indicate that, worldwide, more than 50 million people suffer from dementia, with approximately 70% being attributed to Alzheimer’s Disease (AD) as the most common form of age-related dementia (Reitz et al., 2011; Prince et al., 2013; Nichols et al., 2022). With age being one of the most relevant risk factors for AD and considering the worldwide increases in average life expectancy, dramatical increases can be expected in the time to come. Next to age and other unmodifiable risk factors such as gender and genetics, a number of modifiable risk factors have been identified that may influence the incidence and clinical course of AD, including physical activity, classic cardio-/cerebrovascular risk factors (i.e., arterial hypertension, diabetes, smoking) and adiposity (Edwards et al., 2019). Considering the fact that approximately 68% of the US adult population are estimated to be overweight (2016; BMI > 25 kg*m^–2^)^1^ further underscores the necessity to promote our understanding of the relationship between body weight and AD.

However, to date, controversy exists on how overweight and obesity affect the risk for AD and its clinical course. A number of studies reported a higher risk for future AD in middle aged overweight/obese subjects. In contrast, elevated body weight has been found to have protective properties in elderly subjects leading to the description of the “obesity paradox” in AD (Emmerzaal et al., 2015). Importantly however, several studies found dynamic changes of body weight—even in preclinical stages–indicating an early interference of AD with homeostatic functions (Gu et al., 2014; Alhurani et al., 2016; Cova et al., 2016).

While potential biological mechanisms of the AD obesity paradox remain elusive, adipokines—and most prominently leptin–have been extensively discussed in the context of body weight and AD. Being secreted by adipocytes, leptin concentrations rise proportionally with fat mass and its putative functions—next to a major role in energy homeostasis—include potential neuroprotective properties (Paz-Filho et al., 2010; McGuire and Ishii, 2016).

Several neuroimaging studies have attempted to investigate the link between AD and body weight. Here, similar to epidemiological studies mixed results have been obtained. At the structural and anatomical aspect, widespread negative associations have been found in AD and MCI subjects (Ho et al., 2010; Boyle et al., 2015) with body weight. On the other hand, support for the existence of a potentially beneficial effect of higher body weight on AD pathology has been provided by more recent studies investigating PET-based measures of amyloid-β pathology (Hsu et al., 2016; Thirunavu et al., 2019). However, considering current models of AD pathophysiology, crucial upstream pathophysiological events such as Aβ deposition may have already peaked in preclinical stages (Jack et al., 2010). In contrast, downstream pathophysiological events such as brain metabolic reductions–as measured by FDG PET–may provide a more sensitive marker of disease progression (Alexander et al., 2002; Reiman and Jagust, 2012). Uptake of the PET tracer [18F]fluorodeoxyglucose (FDG) is directly proportional to local glucose consumption, as both glucose and FDG are being delivered to brain cells *via* identical transport mechanisms. Cerebral glucose metabolism on the other hand depends on local neuronal function and activity, which is why FDG uptake has been extensively used as non-invasive method for the estimation of the regional cerebral metabolic rate of glucose utilization (rCMRgl) in the study of AD and potential risk factors (Reiman et al., 2008, 2010; Langbaum et al., 2009, 2010; Burns et al., 2013; Sabbagh et al., 2015).

Thus far, few studies have investigated the effect of body weight and adiposity on brain glucose metabolism in both cognitively healthy and symptomatic subjects. Somewhat reflective of epidemiological and non-imaging data, results were incongruent with both potentially detrimental and beneficial effects of a higher BMI on the level of cerebral glucose metabolism (Sugimoto et al., 2017; Blautzik et al., 2018; Malpetti et al., 2018; Sala et al., 2019), although comparability is limited due to the use of both region of interest and whole brain approaches. Importantly however, all of these studies were restricted to cross-sectional data, hence it remains unknown how body weight is related to longitudinal changes of brain glucose metabolism.

We therefore aimed to investigate the relationship of BMI with rCMRgl and its longitudinal declines in a large clinical population with a diagnosis of amnestic mild cognitive impairment (aMCI), hypothesizing that a higher baseline BMI would be related to greater rCMRgl and less longitudinal decline within AD typical brain regions. We performed voxel based analyses of FDG-PET imaging data in a sample of *N* = 716 subjects (cross-sectional) and a subsample (*N* = 453) with available follow-up FDG imaging data. Under consideration of the previously proposed neuroprotective properties of the adipokine leptin we additionally explored the brain metabolic correlates of both plasma and CSF leptin concentrations in further subsamples.

## Subjects and methods

All data were downloaded from the LONI ADNI data repository.^2^ ADNI is a large scale ongoing multicentric longitudinal study with the goal to identify biological and neuroimaging biomarkers of MCI and Alzheimer’s disease. Cross-sectional FDG PET data were downloaded for *N* = 716 aMCI subjects with baseline anthropometric data (i.e., body weight and height). Of these subjects, *N* = 453 had additional follow-up scans (≥6 months). Within the cross-sectional sample, data on plasma and CSF leptin concentrations (measured at baseline; log-transformed) were available for *N* = 202 (plasma leptin) and *N* = 81 (CSF leptin) subjects. Baseline BMI was calculated as: (weight in kg)*(height in m)^–2^. Additional detailed information on recruitment, inclusion/exclusion criteria and sampling methods we refer to the ADNI website.^3^

### Imaging procedures

Alzheimer’s disease neuroimaging initiative offers both unprocessed and preprocessed neuroimaging data. For our study of cross-sectional and longitudinal imaging data from different sites participating in the ADNI trial, we chose the PET data fully preprocessed by the ADNI PET Coordinating Center at the University of Michigan to account for site specific differences in terms of attenuation correction and image resolution including spatial smoothing with a three-dimensional Gaussian filter with 8 mm full width at half maximum and intensity normalization *via* global mean correction, consistent with previous BMI related and ADNI based publications (Malpetti et al., 2018; Sala et al., 2019). Our additional preprocessing steps were performed using SPM8 next, subtraction images of longitudinal FDG scans were calculated (i.e., baseline—follow-up) for characterization and comparisons of longitudinal rCMRgl declines. All images underwent a careful quality check prior to analyses. Please note that in this study rCMRgl refers to the standardized uptake value ratio (SUVR; normalized for whole brain FDG uptake) as a proxy of the cerebral metabolic rate for glucose.

### Cognitive evaluation

In our study, for correlational analyses of cognitive performance and its longitudinal decline we applied the modified AD Assessment Scale-Cognitive Subscale (i.e., ADAS-cog 11; Rosen et al., 1984; Mohs et al., 1997). The ADAS-cog 11 provides a detailed measure of multiple cognitive domains including memory/new learning and language. For the ADAS-cog 11 higher scores indicate worse performance. For longitudinal data, delta values were calculated as “follow-up minus baseline” (i.e., positive values indicating cognitive decline). Additional measures of the ADNI sample—amongst others—include: The Auditory Verbal Learning Test (AVLT) (Rey, 1964), Total and Long-Term Memory (LTM) scores, CDR sum of boxes (CDR-SOB) (Morris, 1993), MMSE (Folstein et al., 1975), and Category Fluency (animals) (Rosen, 1980).

### Image data analysis

Final data analyses were carried out using Statistical Parametric Mapping 12 (SPM12; Welcome Department of Cognitive Neurology, London, UK). Voxel-wise analyses were conducted using multiple regression models to examine associations of baseline BMI with rCMRgl in the entire sample of aMCI subjects. Here, different models were applied to investigate the impact of different variables. First simple models were carried out adjusting for baseline age and gender only. Further models included additional adjustments for carriers of the ε4 allele (binary yes/no) or global cognition as measured by the ADAS-cog 11 as a continuous variable. Longitudinal analyses were performed analogously with the correction for follow-up time. Multiple regression analyses of plasma and CSF leptin concentrations with rCMRgl also included different models: A simple model with age and gender as covariates of no interest and a full model with additional adjustments for ε4 carriers and BMI, in order to account for the positive association between BMI and leptin, thus allowing to investigate leptin-specific effects. For all analyses we used a standard whole brain gray matter mask to reduce the risk of artifacts. Tables with corresponding detailed results are provided as Supplementary Data Sheet 1.

Additional *Post-hoc* analyses regarding differential associations of BMI and leptin concentrations in ε4 carriers vs. non-carriers were investigated by comparing slope strengths of the respective variable of interest (i.e., ε4*BMI, ε4*Leptin_Plasma_ and ε4*Leptin_CSF_ interaction) using SPM’s ANOVA. Corresponding detailed results (figures and tables) are provided as Supplementary Data Sheet 1.

For all analyses we made use of the Threshold Free Cluster Enhancement (TFCE) toolbox^4^ which provides robust permutation based non-parametric statistics and does not require to prespecify any arbitrary thresholds. Results were considered significant at *p* < 0.05 FWE_TFCE_ whole brain corrected after 5,000 permutations (E = 0.6). For ANOVA based interaction analyses results are illustrated and reported at a more exploratory threshold of *p* < 0.1 FWE_TFCE_. MRIcron software^5^ was used for illustration generations.

For non-imaging analyses we used SAS statistical software (SAS 9.3 and Enterprise Guide 5.1).

## Results

Population characteristics are illustrated in Table 1. aMCI subjects carrying the ε4 allele were modestly lighter weighted as compared to non-carriers (*p* < 0.02). Cross-sectionally, BMI was not significantly related to cognitive performance (i.e., ADAS-cog 11; rho = –0.06, *p* = 0.1). Longitudinally however, a higher baseline BMI was associated with less cognitive decline (i.e., ΔADAS-cog 11; rho = –0.11, *p* = 0.02).

**TABLE 1:** Population characteristics.

**Cross-sectional sample (*N* = 716)**	
Gender (m/f)*	420/296
Age (years)	73.0 ± 7.7
Baseline BMI (kg*m^–2^)	27.3 ± 4.9
ε4 carriers (*N* = 711; y/n)	342/369
ADAScog11 (*N* = 712)	9.7 ± 4.3
Plasma leptin LOG (*N* = 202; ng/ml)	0.90 ± 0.41
Log CSF leptin LOG (*N* = 81; ng/ml)	–1.02 ± 0.31
**Longitudinal subsample (*N* = 453)**	
Time interval (days)	1,192 ± 733
Gender (m/f)	273/180
Age (years)	72.9 ± 7.6
Age (years)	72.9 ± 7.6
Baseline BMI (kg*m^–2^)	27.1 ± 4.9
ε4 carriers (y/n)*	208/245
ΔADAScog11 (*N* = 444)	3.5 ± 7.4

Population characteristics are presented as mean ± standard deviation except for *.

### Cross-sectional associations of body mass index with regional cerebral metabolic rate of glucose

**FIGURE 1:**
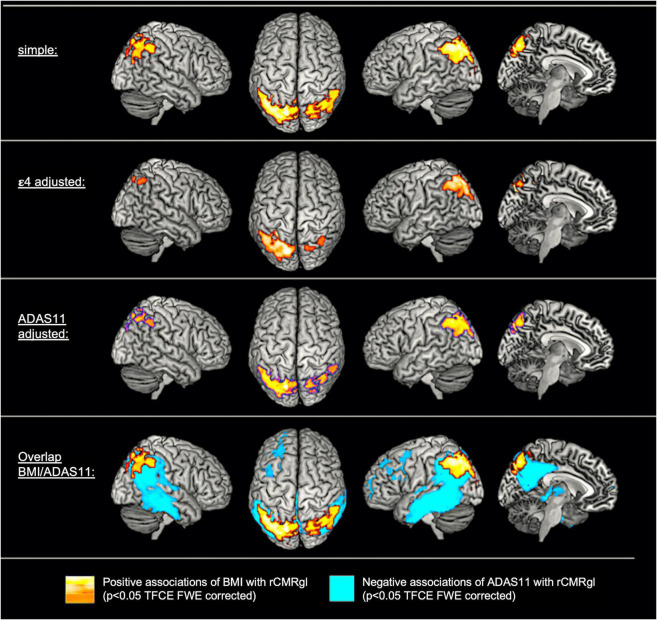
Cross-sectional associations of body mass index (BMI) and cognitive function with regional cerebral metabolic rate of glucose (rCMRgl) in subjects with amnestic mild cognitive impairment (MCI). For exemplary scatter plots see Supplementary Figure 2.

**FIGURE 2:**
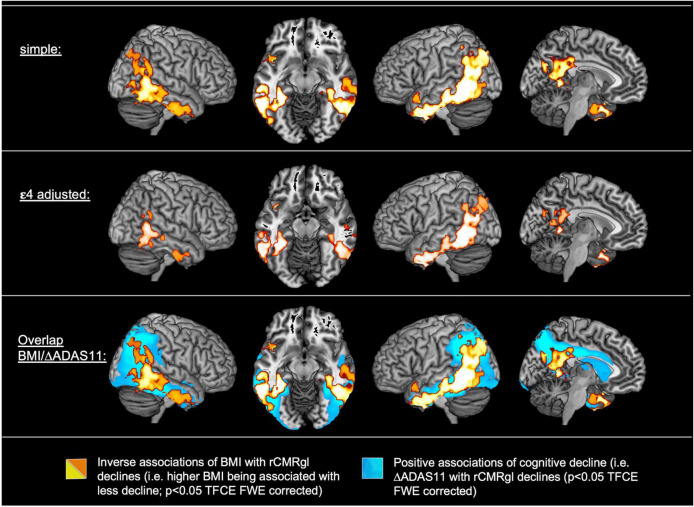
Associations of baseline body mass index (BMI) (*N* = 453) and cognitive decline (ΔADAS-cog 11; *N* = 444) with longitudinal regional cerebral metabolic rate of glucose (rCMRgl) declines in subjects with amnestic mild cognitive impairment (MCI). For exemplary scatter plots see Supplementary Figure 2.

For detailed results please see Figure 1 and Supplementary Table 1. Simple models adjusted for age and gender yielded positive associations (*p* < 0.05 TFCE FWE whole brain corrected) of BMI with rCMRgl within parietooccipital brain region, most significantly within the bilateral precuneus. Additional adjustment for cognitive function (i.e., ADAS-cog 11) did not significantly change these results. Adjustment for ε4 carriers yielded a similar yet less significant pattern of bilateral associations within the parietal lobe (left > right). Worse cognitive function on the other hand–as measured by the ADAS-cog 11—was associated with lower rCMRgl within largely overlapping brain regions (illustrated in Figure 2), including the bilateral posterior cingulate, the bilateral lateral and medial temporal lobes, the bilateral parietal lobes (i.e., bilateral precuneus) and left frontal brain regions.

In addition, a significant ε4*BMI interaction term was observed within frontal cortical regions, including the bilateral cingulate gyrus and the right middle, medial and superior frontal gyrus, with BMI being positively associated with rCMRgl in ε4 non-carriers and negatively associated in ε4 carriers (for detailed results see Supplementary Figure 1 and Supplementary Table 4).

Additional *post-hoc* adjustments for plasma glucose concentrations (*N* = 711) did not significantly change our main results (data not shown).

### Associations of baseline body mass index with longitudinal regional cerebral metabolic rate of glucose declines

Detailed results are illustrated in Figure 2 and Supplementary Table 2. Simple models adjusted for age, gender and TI yielded extensive inverse associations (*p* < 0.05 TFCE FWE whole brain corrected) of baseline BMI with longitudinal rCMRgl declines (i.e., higher baseline BMI being associated with less rCMRgl decline) within the bilateral posterior cingulate, the bilateral lateral and medial temporal lobe and the parietal lobes (including the bilateral precuneus). Adjustment for ε4 carriers yielded a similar yet less extent pattern of inverse associations. Simple models additionally yielded positive associations of BMI with rCMRgl declines (i.e., higher baseline BMI being associated with stronger rCMRgl decline) within the left precentral gyrus. However, when adjusted for cognitive decline (i.e., ΔADAS-cog 11) none of these associations remained significant, whereas cognitive decline was associated with widespread and highly significant rCMRgl declines within similar AD-typical brain regions (overlap illustrated in Figure 2). No significant ε4*BMI interaction term was observed (all *p* > 0.1 TFCE FWE whole brain corrected).

Additional *post-hoc* adjustments for plasma glucose differences (*N* = 446) did not significantly change our main results (data not shown).

### Associations of plasma or cerebrospinal fluid leptin with regional cerebral metabolic rate of glucose

**FIGURE 3:**
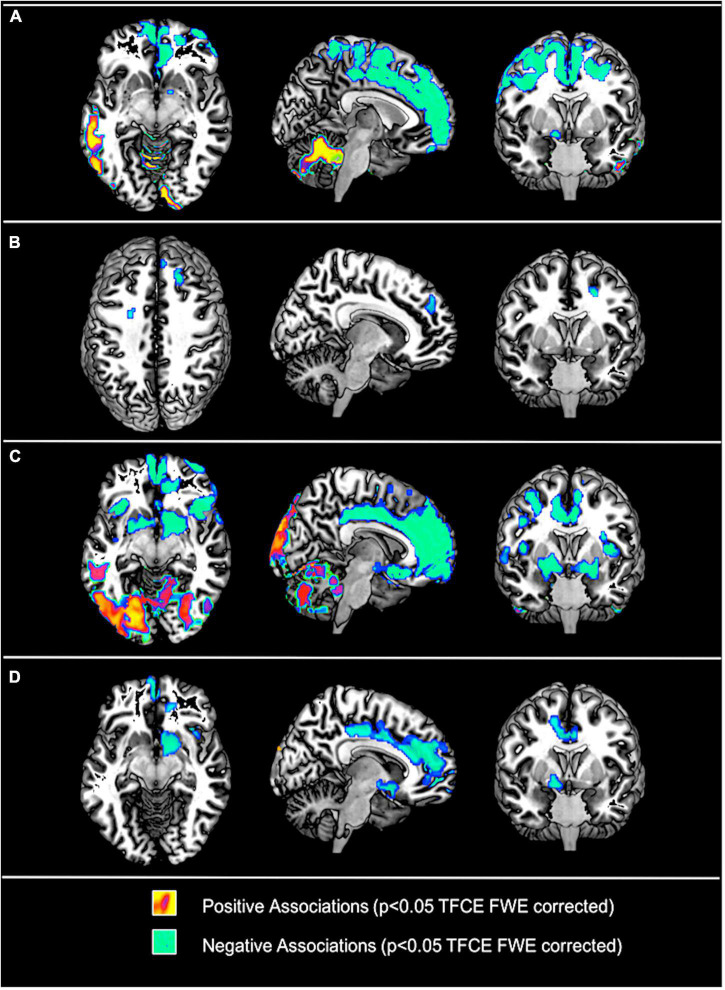
Associations of plasma leptin concentrations without **(A)** and with **(B)** and cerebrospinal fluid (CSF) leptin concentrations without **(C)** and with **(D)** adjustments for body mass index (BMI) and ε4 respectively with regional cerebral metabolic rate of glucose (rCMRgl) in subjects with amnestic mild cognitive impairment (MCI). Results are illustrated at *p* < 0.05 threshold free cluster enhancement (TFCE) whole brain corrected (FWE). Cool colors illustrate negative association, warm colors illustrate positive associations.

For detailed cross-sectional results please see Figure 3 and Supplementary Table 3. Expectedly, plasma and CSF leptin concentrations were each positively associated with BMI (plasma: rho = 0.70; *p* < 0.001; CSF: rho = 0.67: *p* < 0.001; partial correlations adjusted for age and gender). Uncorrected for BMI, plasma leptin concentrations yielded a distinct pattern of both positive and negative associations with positive associations being located within the cerebellum and the parietooccipital cortex, whereas, extensive negative associations were found within the frontal cortex–most significantly within the medial prefrontal cortex (mPFC)—and the right ventral striatum. After additional correction for BMI, only negative associations remained significant within the bilateral medial and lateral PFC. A similar, yet more pronounced pattern was observed for CSF concentrations (unadjusted for BMI) with positive associations within the cerebellum and parietooccipital cortical regions and extensive negative associations within the medial and lateral prefrontal cortex, the bilateral insula and the bilateral dorsal and ventral striatum. After additional adjustment for BMI, CSF leptin concentrations were still positively associated with occipital and cerebellar regions (although less extent) and negatively associated with medial cortical regions including the medial prefrontal gyrus together with more dorsal aspects of the cingulate gyrus and the right dorsal and ventral striatum expanding toward the right anterior and posterior insular cortex and the hypothalamus. In contrast, BMI was not significantly associated with rCMRgl when controlled for plasma and CSF leptin concentrations respectively. Of note, when BMI was analyzed individually in both subsamples (i.e., without adjusting for leptin; data not shown), no significant associations were found for BMI in the CSF subsample (*N* = 81). In the plasma subsample (*N* = 202), positive (with cerebellar rCMRgl) and negative (with right prefrontal rCMRgl) associations were seen for BMI that closely resembled our associations with leptin, but were much less extent. With respect to positive associations observed in the entire sample (*N* = 716) of aMCI participants, overlap within the left parietal cortex was only seen at more liberal thresholds (i.e., *p* < 0.1 FWE TFCE whole brain corrected).

For longitudinal declines of rCMRgl no significant (*p* < 0.05 FWE_TFCE_) associations were found for both plasma and CSF plasma concentrations.

Significant ε4*leptin interaction terms (for detailed results see Supplementary Figure 1 and Supplementary Table 4) were found for plasma leptin concentrations within the left temporal cortex (carriers > non-carriers) and the right frontal cortex (non-carriers > carriers). For CSF leptin concentrations a significant ε4*leptin interaction term was found within cerebellar/occipital regions (non-carriers > carriers).

Additional *post-hoc* adjustments for plasma glucose differences (*N* = 201 and *N* = 81 respectively) did not significantly change our main results (data not shown).

## Discussion

Here we investigate the relationship of BMI with rCMRgl (as estimated by PET FDG uptake) and its longitudinal declines in a large clinical population diagnosed with aMCI. Additionally we explored the relationship between plasma and CSF concentrations of the adipokine leptin with cerebral glucose metabolism. Briefly summarized, a higher baseline BMI was associated with higher rCMRgl in a sample of *N* = 716 aMCI patients (cross-sectional). These associations were largely limited to heteromodal parietal association areas (i.e., bilateral precuneus) that are typically affected by AD and showed a near-complete overlap with rCMRgl correlates of cognitive function. In a subsample of *N* = 453 subjects with follow-up data a comparable yet more extent pattern of inverse associations of baseline BMI with rCMRgl declines (i.e., higher BMI being associated with less rCMRgl decline) was observed. Again, these associations were confined to AD-typical brain regions and showed strong overlap with the correlates of longitudinal cognitive decline. However, while cross-sectional results were independent of cognitive function, our longitudinal analyses of BMI did not remain significant when controlling for cognitive decline. For plasma and CSF concentrations of the adipocyte derived hormone leptin, we show a bidirectional pattern of positive associations with cross-sectional rCMRgl, restricted to parietal, occipital, temporal (in parts) and cerebellar regions and negative associations within prefrontal/limbic and subcortical structures. However, after adjustment for BMI, leptin concentrations were almost exclusively negatively associated with mesocorticolimbic brain regions implicated in the regulation of energy homeostasis and longitudinal analyses did not yield significant associations on the whole-brain level. Additional *post-hoc* analyses only showed subtle differences between carriers and non-carriers of the APOE ε4 risk allele for associations of BMI and leptin concentrations with brain glucose metabolism.

Epidemiological data provide support for our cross-sectional and longitudinal observations. Higher BMI In older subjects has been repeatedly found to be associated with a decreased risk for dementia and AD, while on the other hand several studies found middle aged overweight or obese subjects to have an increased risk for future AD (Chuang et al., 2015; Emmerzaal et al., 2015). Nevertheless, longitudinal data in close to two million subjects showed similar effects for both middle and old aged subjects, with BMI being negatively associated with the risk for dementia (Qizilbash et al., 2015), overall indicating a reduced risk in overweight and obese subjects regardless of age. However, as specific diagnoses were not available, the latter study was not able to differentiate specific forms of dementia (i.e., AD vs. vascular dementia, Lewy Body Dementia etc.). These—in parts contradictory–findings may be explained by results obtained by multiple trajectory studies that demonstrated a greater risk for the development of dementia and AD in subjects with declining BMI in later adulthood (Gu et al., 2014; Emmerzaal et al., 2015; Alhurani et al., 2016; Cova et al., 2016), a relationship that appears to be further modified by the APOE genotype with carriers of the ε4 allele exhibiting a steeper decline of BMI (Bäckman et al., 2015). Our data provide some support for these observations, as we found a significantly lower BMI in carriers of the ε4 allele. For our cross-sectional analyses we also found a significant ε4*BMI interaction term with a stronger positive association for BMI with rCMRgl in non-carriers of the ε4 allele. Nevertheless, these results were restricted to AD untypical prefrontal brain regions and additional adjustments for ε4 carriers in both cross-sectional and longitudinal analyses did result in highly similar patterns of associations.

Previous studies have investigated the associations of body weight with neuroimaging biomarkers of AD. Here, somewhat similar to epidemiological studies, conflicting results have been observed. Earlier structural MRI (sMRI) studies have found increased mesial temporal atrophy in AD patients with low body weight (Grundman et al., 1996) while obesity has been shown to be associated with brain volume deficits within the frontal, occipital, temporal and parietal lobes in a sample of MCI and AD patients (Ho et al., 2010). Similar results were observed in separate large (*N* = 963) cross-sectional study of patients diagnosed with AD or MCI and cognitively healthy subjects (Boyle et al., 2015). Nevertheless, cautious interpretation of these results is required in the context of Alzheimer’s disease and dementia, as an increasing number of studies found overweight and obesity to be associated with structural alterations of similar brain regions, even in cognitively healthy young adults, adolescents and children (Alosco et al., 2014; Ou et al., 2015; Weise et al., 2017). Hence, sMRI based biomarkers may not be a sufficiently specific measure to investigate the interaction of body weight with AD. FDG PET measurements of the cerebral metabolic rate of glucose (rCMRgl)–as applied in this study–have been proven helpful in both establishing diagnosis and tracking the clinical course of AD (Reiman and Jagust, 2012). Given that rCMRgl measures have been shown to predict future AD in MCI patients (Chételat et al., 2003; Chen et al., 2011), our results are potentially consistent with a protective effect of higher BMI in the context of AD. While FDG PET measures of rCMRgl declines represent a rather unspecific surrogate of neurodegeneration, it seems noteworthy that higher body weight has been found to be associated with lower PET measures amyloid-β in older asymptomatic adults (Hsu et al., 2016; Thirunavu et al., 2019). However, AD related amyloid pathology is thought to reach a plateau, thus not representing a sensitive measure of disease progression in MCI or dementia stages, whereas FDG PET measures of brain glucose metabolism may provide a superior imaging biomarker of disease progression (Khosravi et al., 2019).

On the other hand, brain metabolic reductions may be influenced by a partial volume effect due to regional brain atrophy, which has not been corrected for in this study. Since BMI and adiposity have been repeatedly shown to be associated with brain structural alterations in healthy and diseased populations, this may have affected our results. Nevertheless, previous studies found AD-related hypometabolic patterns not to be significantly affected by atrophy (e.g., Reiman et al., 2004; Bejanin et al., 2019) and the vast majority of studies investigating the relationship between brain structure and obesity found inverse associations of brain structural/volumetric features with body weight (e.g., Ho et al., 2010) which in turn are unlikely to explain our positive associations between BMI and rCMRgl estimates (both cross-sectionally and longitudinally).

Thus far, no conclusive explanation exists for the suggested protective properties of higher body weight in the context of dementia and AD, particularly since obesity related comorbidities, such as hypertension, diabetes and hyperlipidemia have been identified as additional risk factors for AD (Chui et al., 2012; Edwards et al., 2019). Nevertheless, it is possible that adipose tissue and its endocrine properties might have beneficial effects with respect to neurodegenerative processes. The adipokine leptin is predominantly secreted by adipocytes, with plasma concentrations rising in proportion to fat mass. Its primary function is to regulate energy homeostasis and metabolism by providing a humeral signal of available energy reserves *via* the arcuate nucleus of the hypothalamus (Gautron and Elmquist, 2011). Yet evidence points to a much more diverse role, that among others include immune function (Lago et al., 2008), bone metabolism (Upadhyay et al., 2015), neurogenesis and synaptic function (Paz-Filho et al., 2010). Its pleiotropic extrahypothalamic central actions and potential neuroprotective properties include the modulation of AD specific pathophysiological mechanisms (e.g., reduction of Aβ burden), thus making leptin a pharmaceutical target for the treatment of AD (Paz-Filho et al., 2010; McGuire and Ishii, 2016). Intriguingly, leptin concentrations were associated with a reduced incidence of AD in old adults (Lieb et al., 2009), while replacement therapy in genetically leptin deficient humans was accompanied by increases in regional gray matter volume (Matochik et al., 2005; Paz-Filho et al., 2008; London et al., 2011) and improvement in cognitive abilities, further emphasizing the importance of leptin in terms of neuroplasticity and brain function.

To date, this is the first study to investigate the relationship between plasma and CSF leptin concentrations with cerebral glucose metabolism. Here we found plasma and CSF leptin concentrations to be positively associated with cross-sectional rCMRgl of cerebellar and posterior aspects of the cerebral cortex including the temporal, occipital, and parietal cortices. Particularly cerebellar results seem interesting considering previously reported cerebellar implications in the context of leptin signaling and feeding behavior (e.g., Baicy et al., 2007; Zhu and Wang, 2008; Berman et al., 2013). Importantly, human post-mortem studies showed—next to the hypothalamus–highest expressions of the long form of the leptin receptor in the cerebellum (Burguera et al., 2000) potentially providing a biological explanation for the here observed regional accentuation. Nevertheless, positive associations nearly completely disappeared after additional adjustment for BMI and no significant associations were observed for leptin measures with longitudinal rCMRgl declines.

On the other hand, we found extensive negative associations of both plasma and CSF leptin with cross-sectional rCMRgl of prefrontal and subcortical structures including the dorsal and ventral striatum, the insular cortex and parts of the hypothalamus. Additional adjustment for BMI attenuated these associations, however, the pattern of involved frontal and subcortical regions remained unchanged, particularly for CSF measures of leptin. Interestingly, these brain regions are strongly implicated in the neurobiology of homeostatic functions (Craig, 2002; Waterson and Horvath, 2015). Considering leptin’s primary function–providing a humoral signal of one’s energy reserves–these findings may indeed rather reflect physiological changes of brain glucose utilization within brain regions implicated in energy homeostasis rather than disease specific alterations. Indeed, studies in leptin deficient subjects showed marked changes within mesolimbic brain regions (i.e., Nacc) in response to visual food stimuli after leptin treatment (Farooqi et al., 2007), however, further research including healthy subjects is required to validate our findings and gain further insight on the relationship between leptin and brain metabolism. Nevertheless, with respect to the relationship between leptin and brain glucose metabolism, differences were observed between carriers and non-carriers of the AD risk modifying ε4 allele in our study. Although, these differences were subtle and not restricted to AD typical brain regions, our findings potentially do indicate that central leptin signaling may interact with APOE ε4 related brain metabolic disturbances. Interestingly, the LDLR-related protein 1 (LRP1) has been shown to interact with both APOE ε4 mediated Aβ-pathology and leptin signaling (Shinohara et al., 2017), thus providing a common link for both pathways and a potential target for future research in the context of the intricate relationship between AD, adiposity and energy metabolism. In this regard however, we have to acknowledge that the human nutritional status is complexly regulated, and alterations such as overweight and obesity are accompanied by a number of (patho-)physiological adaptations (e.g., vascular risk factors and metabolic-endocrine changes) all of which may influence our results. In contrast to our results however, previous reports regarding vascular risk factors such as hypertension, hypercholesterolemia, type 2 diabetes and prediabetes overall indicated a negative impact on the level of brain metabolism (e.g., Li et al., 2016; Souza et al., 2020; Zhou et al., 2020; Sundermann et al., 2021), making it unlikely that our results and AD’s obesity paradox in general can be ascribed to these factors. Of note, plasma glucose concentrations have been shown to influence FDG estimates of rCMRgl (e.g., Burns et al., 2013; Ishibashi et al., 2015; Sarikaya et al., 2020) and were associated with BMI in our sample of MCI participants. However, glucose adjusted additional *post-hoc* analyses did not indicate a significant impact on our main results. As further limitation we acknowledge that rCMRgl has been estimated *via* FDG uptake (i.e., SUVR) without additional implementation of an input function (e.g., *via* arterialized venous plasma samples or image-derived), since the respective prerequisite data are not provided by ADNI. Additional limitations of this study include the use of BMI as an estimate of adiposity. Overweight and obesity are characterized by increases in both fat mass (FM) and fat-free mass (FFM; i.e., all non-adipose tissues). Although BMI is the most frequently used surrogate for adiposity, no inferences can be drawn regarding body composition. Interestingly, Burns et al. (2010) demonstrated an accelerated loss of FFM in early AD and its linkage with cognitive decline. Similar results were obtained in a sample of ≈7,100 elderly women, showing an association of low FFM with cognitive impairment (Nourhashémi et al., 2002). In addition, the risk for AD and cognitive decline has been linked to reduced muscle strength (Boyle et al., 2009) further indicating an important role for non-adipose tissues and general fitness in the intricate interplay between body weight and AD. In contrast however, Sugimoto et al. (2017) reported a positive association of fat mass but not fat-free mass with prefrontal rCMRgl, although these associations were limited to medial prefrontal areas. Importantly, aging is generally accompanied by substantial changes of body composition (i.e., decrease in FFM and increase in FM) even in the absence of weight change (Schaap et al., 2013). The high mean age of our population with mostly elderly subjects needs to be acknowledged as additional limitation. While the prevalence of cognitive deficits drastically increases with age this is largely inevitable in clinical populations with a diagnosis of MCI. Nevertheless, broad evidence indicates that higher body weight differentially affects brain function/pathology in middle aged and elderly subjects (Emmerzaal et al., 2015). Although *post-hoc* analyses did not indicate a significant age group<BMI interaction term (<65 years vs. >65 years; data not shown) on the level of cross-sectional and longitudinal, results of this study may not be generalizable to younger populations.

## Conclusion

In this study we found that higher BMI is cross-sectionally associated with higher rCMRgl in subjects with aMCI. Analogously, analyses of longitudinal rCMRgl changes showed less decline in aMCI subjects with a higher baseline BMI. Both cross-sectional and longitudinal associations were confined to brain regions that typically exhibit regional hypometabolism in AD and showed strong overlap with rCMRgl correlates of cognitive performance. These data support a potential beneficial effect of a higher body weight on the level of brain glucose utilization, however, mechanisms remain unclear. Although the adipocyte-derived hormone leptin is thought to exert neuroprotective effects in the context of AD, additional cross-sectional analyses showed mostly negative associations of plasma and CSF leptin concentrations, predominantly located within mesocorticolimbic brain regions implicated in the regulation of energy homeostasis and eating behavior. These observations most likely mirror leptins primary physiological functions, hence, the potential beneficial effects of a higher body mass index seem not to be primarily attributable to leptin concentrations.

## Data availability statement

Publicly available datasets were analyzed in this study. This data can be found here: https://adni.loni.usc.edu/data-samples/access-data/.

## Ethics statement

The ADNI study was approved by the Institutional Review Boards of all of the participating institutions (for a full list of institutions see https://adni.loni.usc.edu/wp-content/uploads/how_to_apply/ADNI_Acknowledgement_List.pdf?). The patients/participants provided their written informed consent to participate in this study.

## Author contributions

CW: study design, data analysis, interpretation of data, and manuscript preparation. KC: data analysis, interpretation of data, and critical revision of the manuscript. YC, VD, and YS: data preparation and processing and critical revision of the manuscript. ER: interpretation of data and critical revision of the manuscript. All authors contributed to the article and approved the submitted version.

## Footnotes

1
www.WHO.int

2
https://adni.loni.usc.edu/

3
www.adni-info.org/index.php

4
www.neuro.uni-jena.de/tfce/

5
https://people.cas.sc.edu/rorden/mricron/index.html
