# Supplementary material for: Differential impact of body mass index and leptin on baseline and longitudinal positron emission tomography measurements of the cerebral metabolic rate for glucose in amnestic mild cognitive impairment
Source: Front Aging Neurosci. 2022 Nov 22;14:1031189. doi: 10.3389/fnagi.2022.1031189 (PMC9782536; doi:10.3389/fnagi.2022.1031189)
Supplement: Supplementary file 1 [file Data_Sheet_1.pdf]

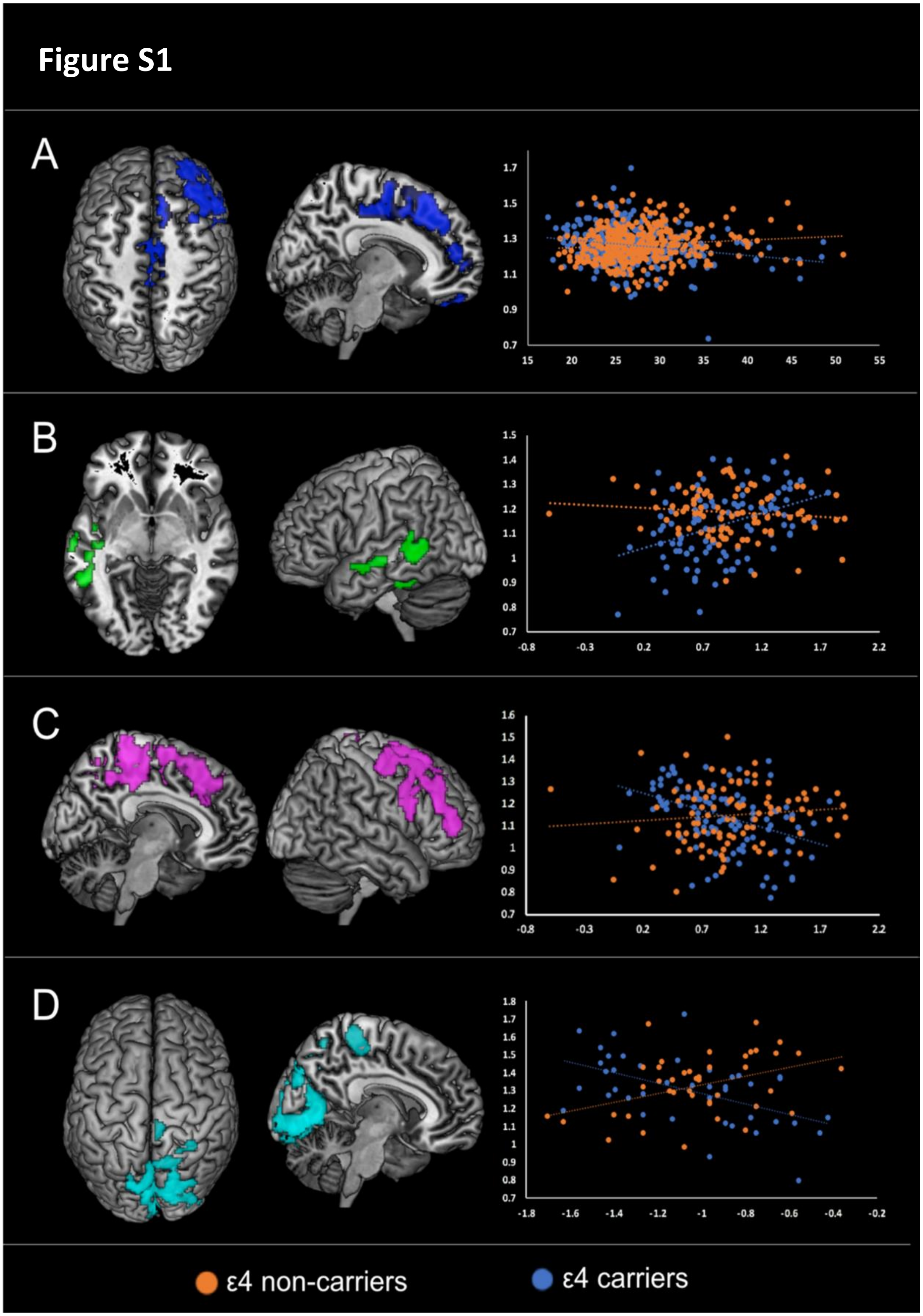

Figure S1: Statistical maps of rCMRgl (illustrated at p<0.1 TFCE FWE whole brain corrected) for e4*BMI (A: non-carriers<carriers; N=711;), e4*plasma leptin (B: carriers>non-carriers; C: non-carriers<carriers; N=202) and e4*BMI CSF leptin (D; non-carriers<carriers; N=81) with the corresponding scatterplots of extracted peak data.

Table S1: Cross-sectional associations of BMI and cognitive function with rCMRgl in subjects with amnestic MCI.

Model	Brain Region	k	p(FWE$_{TFCE}$)	x,y,z {MNI}
**Positive associations of BMI with rCMRgl:**				
Simple model (N=716):*	**L Precuneus[S]**	**2626**	**0.001**	**-16 -76 46**
	L Inferior Parietal Lobule		0.002	-46 -60 46
	L Precuneus		0.002	-18 -68 54
	**R Precuneus**	**1451**	**0.010**	**14 -70 52**
	R Precuneus		0.010	36 -64 48
	R Precuneus		0.011	22 -72 50
	**R Superior Parietal Lobule**	**1**	**0.048**	**40 -50 58**
	**L Precuneus**	**21**	**0.048**	**-2 -58 60**
	**L Cuneus**	**20**	**0.049**	**-12 -94 12**
	**L Middle Occipital Gyrus**	**22**	**0.049**	**-26 -92 10**
	**L Middle Occipital Gyrus**	**2**	**0.050**	**-34 -86 4**
Additional ε4 adjustment (N=711):	**L Precuneus**	**1458**	**0.006**	**-16 -76 46**
	L Precuneus		0.008	-18 -68 54
	L Superior Parietal Lobule		0.009	-30 -64 52
	**R Precuneus**	**89**	**0.040**	**14 -70 54**
	R Precuneus		0.044	22 -72 52
	**R Precuneus**	**81**	**0.042**	**36 -64 48**
Additional ADAS11 adjustment (N=712):	**L Precuneus**	**2196**	**0.002**	**-16 -76 46**
	L Precuneus		0.003	-18 -68 54
	L Superior Parietal Lobule		0.004	-28 -64 52
	**R Precuneus**	**806**	**0.015**	**14 -70 54**
	R Precuneus		0.017	36 -64 48
	R Precuneus		0.017	22 -72 50
	**R Inferior Parietal Lobule**	**99**	**0.038**	**54 -48 44**
**Negative Associations of ADAS-cog 11 with rCMRgl (cross-sectional):**				
Simple (N=712)*	**L Posterior Cingulate**	**21661**	**0.000**	**-4 -56 26**
	L Precuneus		0.000	-4 -70 34
	R Posterior Cingulate		0.000	6 -54 26
	**L Superior Frontal Gyrus**	**820**	**0.030**	**-26 42 42**
	L Superior Frontal Gyrus		0.032	-26 56 2
	L Middle Frontal Gyrus		0.034	-28 32 38
	**L Inferior Frontal Gyrus**	**489**	**0.035**	**-44 10 28**
	L Middle Frontal Gyrus		0.035	-44 18 32
	L Inferior Frontal Gyrus		0.039	-48 20 22
	**L Superior Temporal Gyrus**	**4**	**0.039**	**-52 0 -6**
	**L Middle Frontal Gyrus**	**157**	**0.042**	**-24 14 42**
	**R Cuneus**	**1**	**0.049**	**30 -82 30**

MNI Montreal Neurological Institute.
Results are listed at a threshold of p<0.05 FWE TFCE corrected;
Bold data indicate primary peak within a cluster; Non-bold data indicate secondary peaks.
* adjusted for age and gender; [S] see scatter plot supplementary Figure S2

Table S2: Associations of baseline BMI and cognitive decline with longitudinal rCMRgl declines in subjects with amnestic MCI.

Model	Brain Region	k	p(FWE$_{TFCE}$)	x,y,z {MNI}
**Associations of BMI with longitudinal rCMRgl declines:**				
Simple; neg. (N=453):*	**L Middle Temporal Gyrus**	**10332**	**0.002**	**-50 -50 6**
	L Middle Temporal Gyrus		0.002	-46 -56 12
	L Fusiform Gyrus		0.002	-44 -38 -18
	**R Fusiform Gyrus^{S}**	**6016**	**0.003**	**46 -46 -12**
	R Fusiform Gyrus		0.004	50 -56 -14
	R MiddleTemporal Gyrus		0.005	56 -50 2
Simple; pos. (N=453)*	**L Precentral Gyrus**	**20**	**0.048**	**-28 -12 56**
	L Precentral Gyrus		0.048	-36 -16 60
Additional ε4 adjustment; neg. (N=453):	**L Middle Temporal Gyrus**	**5898**	**0.008**	**-50 -50 6**
	L Fusiform Gyrus		0.008	-44 -38 -18
	L Fusiform Gyrus		0.008	-40 -44 -14
	**R Fusiform Gyrus**	**1450**	**0.009**	**46 -46 -12**
	R Sub-Gyral		0.012	54 -50 -12
	R Middle Temporal Gyrus		0.013	56 -52 2
	**R Parahippocampal Gyrus**	**145**	**0.030**	**20 -48 2**
	R Posterior Cingulate		0.032	22 -54 10
	**R Fusiform Gyrus**	**149**	**0.042**	**62 -12 -32**
	R Inferior Temporal Gyrus		0.045	50 -10 -42
	R Inferior Temporal Gyrus		0.046	56 -6 -36
	**L Parietal Subgyral WM**	**97**	**0.043**	**-30 -46 36**
	L Precuneus		0.046	-26 -46 46
	L Inferior Parietal Lobule		0.050	-40 -48 46
	**R Middle Temporal Gyrus**	**31**	**0.046**	**70 -30 -10**
	**R Superior Temporal Gyrus**	**64**	**0.046**	**58 -46 20**
	R Superior Temporal Gyrus		0.047	52 -48 28
	**R Middle Temporal Gyrus**	**24**	**0.048**	**58 -20 -10**
**Positive Associations of cognitive decline (∆ADAS-cog 11) with longitudinal rCMRgl declines:**				
Simple (N=444):*	**L Superior Temporal Gyrus**	**60241**	**0.000**	**-36 -52 38**
	L Precuneus		0.000	-34 -62 44
	L Temporal Subgyral WM		0.000	-28 -66 34
	**L Superior Frontal Gyrus**	**1707**	**0.008**	**-36 26 50**
	L Middle Frontal Gyrus		0.008	-32 4 66
	L Middle Frontal Gyrus		0.008	-44 26 40
	**R Middle Frontal Gyrus**	**119**	**0.013**	**36 18 58**
	R Middle **Frontal** Gyrus		0.013	40 12 62
	R Middle Frontal Gyrus		0.020	46 12 54
	**L Insula**	**173**	**0.014**	**-38 16 0**
	L Insula		0.015	-32 18 6
	L Claustrum		0.016	-26 26 0
	**R Cingulate Gyrus**	**2**	**0.015**	**14 8 36**
	**L Superior Frontal Gyrus**	**2**	**0.016**	**-8 18 68**

MNI Montreal Neurological Institute.
Results are listed at a threshold of p<0.05 FWE TFCE corrected; *Crb* Cerebellum;
Bold data indicate primary peak within a cluster; Non-bold data indicate secondary peaks.
* adjusted for age and gender and time interval; ^{S} see scatter plot supplementary Figure S2

Table S3: Associations of plasma (N=202) and CSF (N=81) leptin concentrations with rCMRgl (cross-sectional) in subjects with amnestic MCI (N=202).

Model	Brain Region	k	p(FWE_{TFCE})	x,y,z {MNI}
Plasma leptin pos.; simple:*	**L Crb, Anterior Lobe**	**3580**	**0.013**	**-14 -42 -30**
	L Crb, Anterior Lobe		0.018	-6 -46 -26
	R Crb, Uvula		0.021	28 -84 -26
	**L Middle Temporal Gyrus**	**2069**	**0.023**	**-60 -44 -4**
	L Fusiform Gyrus		0.023	-56 -64 -12
	L Crb, Tuber		0.026	-54 -54 -24
	**L Inferior Parietal Lobule**	**94**	**0.045**	**-48 -52 42**
	L Inferior Parietal Lobule		0.047	-54 -42 40
	**R Middle Occipital Gyrus**	**38**	**0.047**	**34 -82 6**
Plasma leptin neg.; simple:*	**R Superior Frontal Gyrus[S]**	**13051**	**0.001**	**22 36 42**
	R Superior Frontal Gyrus		0.001	22 24 58
	R Middle Frontal Gyrus		0.001	38 32 38
*Plasma leptin neg.; full**:*	**L Middle Frontal Gyrus**	**26**	**0.044**	**-24 2 42**
	**R Superior Frontal Gyrus**	**52**	**0.044**	**22 36 42**
	**R Medial Frontal Gyrus**	**34**	**0.046**	**6 48 34**
CSF leptin pos.; simple:*	**L Crb, Declive**	**11691**	**0.002**	**-22 -78 -10**
	L Middle Occipital Gyrus		0.002	-42 -86 16
	L Middle Occipital Gyrus		0.002	-36 -88 10
	**L Crb, Cerebellar Tonsil**	**387**	**0.034**	**-44 -68 -50**
	L Crb, Pyramis		0.035	-26 -88 -32
	L Crb, Cerebellar Tonsil		0.035	-46 -60 -50
	**L Precuneus**	**16**	**0.036**	**-14 -74 34**
	**L Crb, Inferior Semi-Lunar**	**65**	**0.045**	**-20 -82 -48**
*CSF leptin neg.; simple**	**L Cingulate Gyrus**	**13061**	**0.000**	**-6 34 24**
	L Medial Frontal Gyrus		0.000	0 52 4
	L Cingulate Gyrus		0.000	-6 22 30
	**R Precentral Gyrus**	**666**	**0.029**	**36 -2 52**
	R Precentral Gyrus		0.032	52 -8 34
	R Middle Frontal Gyrus		0.033	30 -2 42
	**R Parietal Lobe, Sub-gyral**	**36**	**0.048**	**34 -30 44**
	R Postcentral Gyrus		0.049	34 -28 52
*CSF leptin pos.; full**:*	**L Cuneus**	**12**	**0.049**	**-16 -96 24**
*CSF leptin neg.; full**:*	**L Cingulate Gyrus[S]**	**2524**	**0.010**	**-6 34 24**
	L Medial Frontal Gyrus		0.011	-2 54 4
	L Medial Frontal Gyrus		0.012	-4 42 30
	**R Lentiform Nucleus**	**239**	**0.024**	**10 4 -8**
	R Medial Frontal Gyrus		0.033	18 10 -22
	**R Middle Frontal Gyrus**	**167**	**0.033**	**32 38 32**
	R Superior Frontal Gyrus		0.044	38 44 28
	R Middle Frontal Gyrus		0.045	40 32 22
	**R Middle Frontal Gyrus**	**125**	**0.041**	**32 22 58**
	R Middle Frontal Gyrus		0.043	26 30 54
	R Middle Frontal Gyrus		0.046	30 24 48
	**R Anterior Cingulate**	**54**	**0.042**	**12 44 -4**

MNI Montreal Neurological Institute.
Results are listed at a threshold of p<0.05 FWE TFCE corrected; Crb Cerebellum;
Bold data indicate primary peak within a cluster; Non-bold data indicate secondary peaks.
* adjusted for age and gender; ** adjusted for age, gender, BMI, ε4;
[S] see scatter plot supplementary Figure S2

Table S4: Differential associations of BMI (N=453), plasma (N=202) and CSF (N=81) leptin concentrations with rCMRgl (cross-sectional) in carriers vs. non-carriers of the ApoE ε4 risk allele

Model	Brain Region	k	p(FWE_{TFCE})	x,y,z {MNI}
ε4*BMI (nc>c)	**R Superior Frontal Gyrus**	**1009**	**0.036**	**10 22 56**
	R Medial Frontal Gyrus		0.041	8 18 46
	R Cingulate Gyrus		0.045	8 30 40
	**R Middle Frontal Gyrus**	**1543**	**0.044**	**32 38 40**
	R Middle Frontal Gyrus		0.044	40 34 34
	R Middle Frontal Gyrus		0.045	54 26 18
	**R Medial Frontal Gyrus**	**211**	**0.059**	**6 52 -24**
	L Medial Frontal Gyrus		0.072	-2 60 -16
	R Medial Frontal Gyrus		0.074	4 42 -28
	**R Inferior Frontal Gyrus**	**117**	**0.067**	**56 10 16**
	**L Middle Frontal Gyrus**	**3**	**0.097**	**-14 -2 64**
**ε4*leptin (plasma; nc>c)**	**L Middle Frontal Gyrus**	**5650**	**0.029**	**-26 28 52**
	L Paracentral Lobule		0.032	2 -36 74
	L Medial Frontal Gyrus		0.035	-6 30 46
	**R Precentral Gyrus**	**4**	**0.100**	**20 -22 68**
**ε4*leptin (plasma; c>nc)**	**L Middle Temporal Gyrus**	**449**	**0.044**	**-54 -44 -2**
	L Superior Temporal Gyrus		0.077	-52 -26 -4
	L Middle Temporal Gyrus		0.079	-52 -42 10
	**L Middle Temporal Gyrus**	**135**	**0.077**	**-64 -16 -8**
	L Superior Temporal Gyrus		0.091	-54 -4 -12
	L Superior Temporal Gyrus		0.098	-44 -2 -22
	**L Fusiform Gyrus**	**74**	**0.090**	**-44 -40 -24**
	**L Insula**	**40**	**0.091**	**-44 -18 -8**
**ε4*leptin (CSF; nc>c)**	**R Crb, Declive**	**4214**	**0.017**	**10 -76 -8**
	R Crb, Culmen		0.017	6 -72 -2
	L Cuneus		0.022	-4 -78 20
	**R Paracentral Lobule**	**193**	**0.073**	**8 -30 60**
	**R Sub-Gyral**	**76**	**0.087**	**32 -44 58**

MNI Montreal Neurological Institute.

Results are listed at a threshold of p<0.1 FWE TFCE corrected; *Crb* Cerebellum;

Bold data indicate primary peak within a cluster; Non-bold data indicate secondary peaks.

All models were adjusted for age and gender.

Figure S2

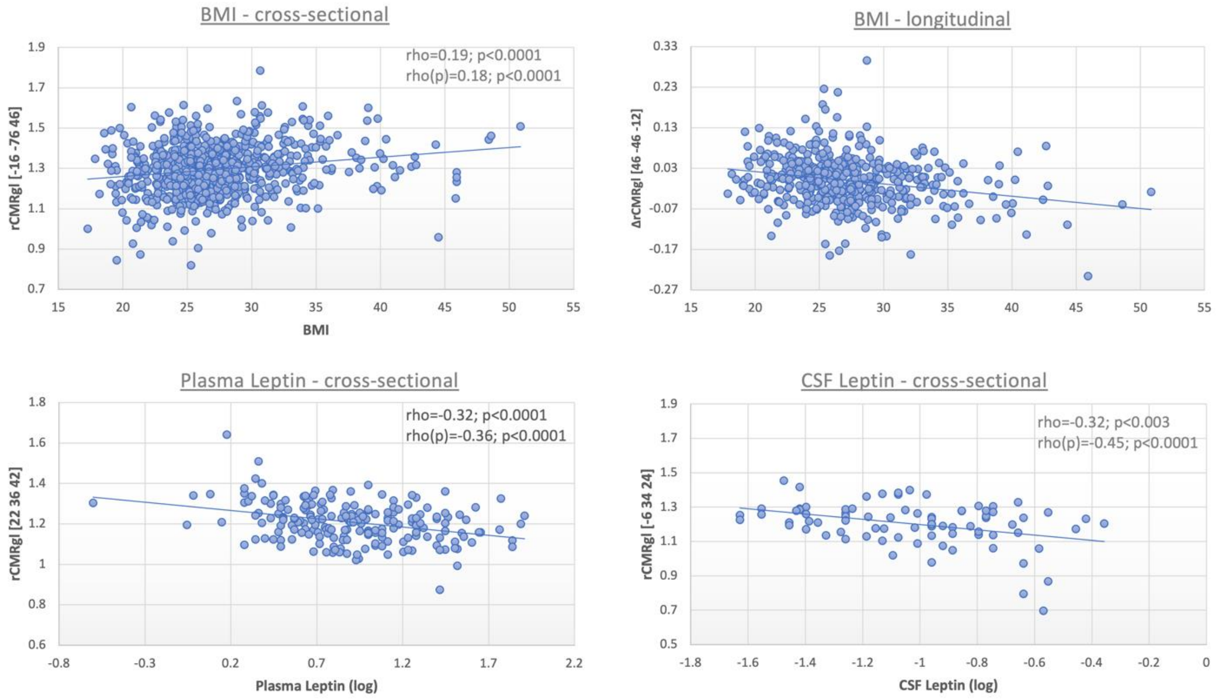

Figure S2: Exemplary scatterplots of the relationships between BMI and leptin (i.e., log-transformed plasma and CSF leptin measures; cross-sectional data only) with cross-sectional rCMRgl and longitudinal ΔrCMRgl declines (i.e., residualized for time interval). Locations (MNI_{xyz}) of extracted voxel data are provided with the y-axis label and are additionally indicated in the corresponding tables. *rho* Spearman correlation coefficient and corresponding p-value without any additional adjustment; rho*(p)* partial Spearman correlation coefficient and corresponding p-value with adjustment for age and gender.
